# Using Vignettes to Gain Insights Into Social Norms Related to Voluntary Family Planning and Gender-Based Violence in South Sudan

**DOI:** 10.9745/GHSP-D-23-00489

**Published:** 2024-06-27

**Authors:** Paul Bukuluki, Moses Okwii, Kamden Hoffmann, Melinda Pavin

**Affiliations:** a Makerere University, Kampala, Uganda.; b Dev-Com Consult Ltd, Juba, South Sudan.; c MOMENTUM Integrated Health Resilience, IMA World Health, Washington, DC, USA.; d MOMENTUM Integrated Health Resilience, John Snow, Inc., Washington, DC, USA.

## Abstract

Social norms are the shared expectations about behaviors that are held within collective groups. These differ from attitudes and beliefs, which are individually held. In South Sudan, social norms can affect the use of voluntary family planning and reproductive health (FP/RH); some of these norms even present risks for women seeking FP/RH care and their FP/RH providers. This cross-sectional, descriptive study conducted in 5 counties in South Sudan used vignettes as a qualitative method to explore social norms related to FP/RH and decisions related to contraceptive use and gender-based violence. Participants were asked to respond to vignettes about a fictional couple during various life stages of care-seeking. Vignettes allow respondents to share their opinions and feelings without directly speaking about their own experiences. Respondents included community members, FP/RH providers, and key influencers (e.g., religious leaders, traditional leaders, elders). The study identified restrictive social norms related to FP/RH, including entrenched, dominant gender roles (i.e., requiring male consent to use contraceptives) and risk for the woman and her provider if she accesses FP/RH services. Of note, supportive social norms are gaining acceptance regarding women’s voice and agency; it was found that some women can negotiate their reproductive choice with their partners and participate in planning their families, which has not always been a widely accepted norm. The use of vignettes can lead to a better understanding of the challenges and provide insights on effective implementation approaches. It is essential for programs working to promote the use of FP/RH services in South Sudan to consider the challenges presented by social norms. Findings from this study were shared with stakeholders and communities to codesign interventions aimed at increasing the use of FP/RH services. Additionally, the dialogue stimulated by this study should lead to an organic transformation toward supportive social norms through collective agency.

## Abstract (precis)

Social norms in South Sudan facilitate or obstruct the use of family planning and reproductive health (FP/RH) services. Understanding these social norms is essential to implementing effective interventions to address barriers to accessing and using FP/RH services.

## Teaser

**Key Findings:**

- Vignettes—short stories about a fictitious South Sudanese couple across the reproductive life course—used to identify social norms related to family planning and reproductive health (FP/RH), provided insight into the social norms that shape some of the attitudes, beliefs, and practices surrounding modern contraceptive use in 5 counties in South Sudan.
- Key restrictive social norms were that women needed consent from their male partners to use contraceptives and, without this consent, women who accessed FP/RH services and their providers were at risk of ridicule, coercion, and violence.
- Supportive social norms included couples practicing postpartum abstinence, girls completing education, and women having agency in FP/RH decisions.

**Key Implication:**

- Program planners should engage with local stakeholders and community members to explore social norms that affect the FP/RH behaviors of populations in their contexts and determine the best approaches for improving the use of FP/RH services and contraceptive use.

## INTRODUCTION

Since South Sudan gained independence from Sudan in 2011, it has remained fragile and unstable, with ongoing humanitarian issues. South Sudan has a young population, with 38% aged 10–24 years.^1^ By age 18 years, 52% are married, contributing to a high adolescent birth rate (158/1,000 girls aged 15–19 years in 2024).^1^ The total fertility rate is 4.1, and maternal mortality ratio is among the highest in the world, estimated at 1,223 deaths per 100,000 births in 2020.^1^

The South Sudan National Family Planning Policy (2013) states that individuals of reproductive age (15 years and older) can access family planning and reproductive health (FP/RH) services. The government of South Sudan made a commitment to the FP 2030 goals of increasing the modern contraceptive prevalence rate (mCPR).^2^ Although these policies and planning frameworks provide opportunities for improving FP/RH, their effective implementation in South Sudan is relatively low, partly due to institutional and resource constraints.

South Sudan core indicators^3^ show modern contraceptive prevalence at 4.2% (mCPR), and unmet need for FP among married women is 30%.

Previous studies on social and cultural norms in South Sudan show that large families had limited support for modern contraception use. These studies had samples limited to single-area and homogeneous study populations but found similar results, including increased social status for both men and women who have large families^4^; a strongly patriarchal society that limits women’s agency and decision-making around contraception^5^^–^^7^; women’s sense of a “national obligation” to replace men lost through war and conflict^8^; men’s strong resentment of women’s use of contraception as a threat to “culture and peoplehood”^9^; and normative associations between the use of contraception and sex outside of marriage or sex work.^9^ Additionally, evidence from sub-Saharan Africa shows that modern contraceptive use is lower among polygamous marriages,^10^ as co-wives compete to produce children.^11^ The polygamy marital structure is common in South Sudan and is often associated with having a stronger sense of social security within the family, clan, and community. Polygamy is accepted for men (i.e., polygyny) but not for women (i.e., polyandry).^12^

Sixty-one percent of respondents on a household survey conducted in South Sudan noted that gender-based violence (GBV) was acceptable under certain circumstances.^13^ Global studies have shown that women who experience intimate partner violence (IPV)—a category of GBV—are more likely to have male partners who refuse to use contraceptives, and they often have little choice about sexual activity.^14^ Reproductive and sexual coercion—a form of IPV—affects women’s agency around FP and is documented across the globe as a factor for unplanned pregnancies, higher rates of abortion, and higher birth rates.^15^^,^^16^

Evidence shows that non-health factors (e.g., education) can be linked to an increased use of modern methods among women and girls.^17^ However, according to some researchers, the specific behaviors that affect girls’ and women’s control over their reproductive health and the social norms relating to connections between IPV and FP are understudied.^16^^,^^18^ Studies have shown that social norms in South Sudan that underlie harmful practices, such as early/child marriage, have negatively affected girls’ education and maternal health—girls often must drop out of school to get married and get pregnant before they are physiologically, emotionally, and socially ready to do so.^19^

Social norms are the implicit and often unspoken “rules” that influence behaviors and practices within a specific group. Unlike attitudes and beliefs, which are individually held, social norms are the mutual expectations about behaviors that are shared within social groups. Social norms are held in place by “reference groups,” defined as “those people whose expectations matter to a given individual in the situation.”^20^ Norms are held in place because of the social rewards for following them and the anticipated negative sanctions for defying them.^20^ Gaining insights into social norms and their key drivers is important for designing FP/RH interventions that are locally appropriate and culturally sensitive to the diverse South Sudanese population.

Gaining insights into social norms and their key drivers is important for designing FP/RH interventions that are locally appropriate and culturally sensitive to the diverse South Sudanese population.

Cislaghi and Heise note that when planning interventions that integrate a social norms perspective, the following 8 learnings should be taken into consideration: (1) social norms and attitudes are different; (2) social norms and attitudes can coincide; (3) protective norms can offer important resources for achieving effective social improvement in people’s health-related practices; (4) harmful practices are sustained by a matrix of factors that need to be understood in their interactions; (5) the prevalence of a norm is not necessarily a sign of its strength; (6) social norms can exert both direct and indirect influence; (7) publicizing the prevalence of a harmful practice can make things worse; and (8) people-led social norm change is both the right and the smart thing to do.^21^ These learnings underscore the complexity of the interactions between social norms and other structural and institutional factors that regulate behavior.^22^

To provide information on commonly held beliefs and attitudes that drive behaviors and practices that affect FP/RH, this study explores social norms related to voluntary FP/RH, as well as issues of GBV, menstrual hygiene and management, and health-seeking behaviors. This study aimed to provide information to major stakeholders for planning programs and developing effective social and behavior change (SBC) activities and messages to improve access to and use of FP/RH care and services for women, girls, and men in South Sudan.

## METHODS

### Study Design

This study was conducted by MOMENTUM Integrated Health Resilience, a U.S Agency for International Development-funded project working to reduce maternal and childhood morbidity and mortality and increase health resilience in fragile settings, including South Sudan.

The study was a cross-sectional, descriptive study that used qualitative methods to explore social norms about “what I think others typically do” (descriptive norms) and about “what I think others expect me to do” (injunctive norms) that facilitate or limit individuals’ and couples’ attitudes and intentions around FP/RH and the use of contraceptives, as well as the risks associated with FP and contraceptive use (e.g., GBV).

Study participants were asked to respond to fictional vignettes about individuals across the life course beginning with sexual debut and marriage through the time a couple has an established family with children. Vignettes are short stories about a person (the protagonist) and/or situation in which respondents can project their experiences and feelings without directly answering questions about themselves or their own experiences.

Vignettes have been documented as useful tools for studying social norms on sensitive topics in specific cultural contexts.^23^^,^^24^ The methods were adapted from the Social Norms Exploration Tool (SNET),^25^ which was developed specifically to support identifying social norms that drive targeted behaviors; the Reproductive Empowerment Scale developed by MEASURE Evaluation^26^; and vignettes in CARE’s Journey Piloting Social Norms Measures for Gender Programming Report.^27^ All vignette questions focused on a fictitious South Sudanese couple and others associated with them.

### Study Sample

The study was conducted in 5 counties in South Sudan: Bor, Budi, Leer, Wau, and Yambio. These counties were identified in collaboration with the U.S Agency for International Development South Sudan Mission. Participants were recruited from selected rural and urban payams (an administrative level just below the county level) in these counties (Table 1). The study population included: (1) community members, comprising women and men aged 18–49 years who were married and unmarried and married women and men aged 15–17 years (South Sudan views married individuals as adults, regardless of age); (2) FP/RH health care providers; and (3) key influencers (e.g., religious leaders, traditional leaders, local chiefs, elders).

**TABLE 1.** Number of Study Participants at Urban and Rural Sites to Explore Social Norms on Voluntary Family Planning and Gender-Based Violence, South Sudan

**County**	**Urban Payams**	**Participants, No.**	**Rural Payams**	**Participants, No.**	**Total**
Bor	Bor	30	Kolnyang, Makuach	25	55
Leer	Leer	37	Pilieny	13	50
Budi	Komori	19	Nagishot, Luodo, Lotukei	33	52
Wau	Wau North, Wau South	42	Besselia	10	52
Yambio	Yambio Town	10	Ri-Rongu, Gangura, Bangasu, Bazungua	47	57
					266

In each study site, women and men of reproductive age were purposely selected from locations that people frequently visited, such as markets and water points. Once identified, prospective respondents were approached to assess eligibility for the study. At health facilities in each study site, we purposively selected health providers who were involved in the provision of FP/RH and GBV-related services. Key influencers were identified through the interviews with community respondents (women and men of reproductive age) and using snowball sampling as influencers identified other influencers. There was 100% response rate. The study planned for 260 respondents, but the actual sample size was 266. There was no opposition to participating in this study (Table 2).

**TABLE 2.** Interviews Planned and Completed by County, South Sudan

**County**	**Community Members, No.**		**Health Providers, No.**		**Key Influencers, No.**	
	**Planned**	**Actual**	**Planned**	**Actual**	**Planned**	**Actual**
Bor	40	43	6	5	6	7
Leer	40	36	6	6	6	6
Budi	40	41	6	6	6	6
Wau	40	40	6	6	6	6
Yambio	40	43	6	9	6	6
Total	200	203	30	32	30	31

In total, 203 community members, 32 FP/RH providers, and 31 key influencers were interviewed (Table 3). Fifty-nine percent of community members were female, whereas 47% of FP/RH providers were female, and 32% of key influencers were female. Twenty-two percent of community members were aged 15–24 years. Thirty-two percent of community respondents and key influencers had no formal education, 30% had some primary education, 13% had some secondary education, and 25% had post-secondary education. Almost half (47%) of the FP/RH providers were nurses; 28% were midwives, 13% doctors, and 13% community health workers.

**TABLE 3.** Characteristics of Participants Interviewed to Explore Social Norms on Voluntary Family Planning and Gender-Based Violence, South Sudan

	**Community Members, No. (%)**	**Health Providers, No. (%)**	**Key Influencers, No. (%)**
Sex			
Female	120 (59)	15 (47)	10 (32)
Male	83 (41)	17 (53)	21 (68)
Age, years			
15–24	44 (22)	0 (0)	0 (0)
25–49	159 (78)	31 (97)	8 (26)
50 and older	0 (0)	1 (3)	23 (74)

### Study Preparation

All data collectors were South Sudanese who spoke at least 1 of the local languages spoken at the study sites. They all had experience in data collection, held at least a bachelor’s degree in public health or a related field, and demonstrated an ability to effectively discuss complex and sensitive issues. Before data collection began, data collectors and field supervisors received training on research ethics, adherence to research protocol, and managing responses and reactions to sensitive topics.

It was important that the vignettes were context specific and expressed realistic scenarios for South Sudan. To do this, drafts of the vignettes were shared with key stakeholders in South Sudan for feedback. The final vignettes were then translated into study site local colloquial languages and prerecorded for use during data collection.

### Data Collection

Data collection occurred from May to June 2021. Interviews with individual study participants were conducted in local languages and were audio-recorded and transcribed for analysis. Five vignettes were used to elicit information on social norms related to FP/RH, beginning with puberty (menstrual hygiene and management), sexual debut, early marriage, and adulthood as a married couple. The vignettes were about Rose and Joe, a South Sudanese couple created for the vignettes, and their relatives: (1) Rose, menstruation; (2) Rose and Joe, a young couple, sexual debut; (3) Theresa (Rose’s cousin), early marriage; (4) Rose, decision to use FP once married with 2 children; (5) Rose, decision-making regarding health care seeking.

The names were adjusted for each site of data collection, selecting common local names for each language group. Respondents were then asked questions about what Rose, Joe, their family, and their community should do in certain situations. Additionally, respondents were given structured statements about Rose and her beliefs and actions and asked if they agreed, disagreed, or did not know. The use of structured statements allowed for quantitative analysis to summarize common norms, while the vignettes allowed for more nuance regarding the norms and led to a broader and richer range of findings.

Female data collectors interviewed women, and male data collectors interviewed men. As needed, interviewers referred study participants to reliable sources of FP/RH and/or GBV services within a reasonable travel distance. Before every interview, the study participant was read a description of the study, information on the risks and benefits of participating in this study, a statement of confidentiality, and a requirement for voluntary participation. Participants gave their consent to be interviewed by providing written consent or a thumbprint if unable to read or write.

Once data were collected, interview recordings were transcribed and translated into English for data analysis.

### Ethical Approval

The protocol for this study was reviewed and cleared by a U.S.-based institutional review board with John Snow, Inc., a partner on MOMENTUM Integrated Health Resilience, and a South Sudan Ministry of Health ethical review board.

### COVID-19 Precautions

Due to the COVID-19 pandemic, additional safety precautions were used to protect the data collectors, study respondents, and others involved in the study. This included the use of personal protective equipment, appropriate social distancing, and conducting interviews outdoors as much as possible. For this reason, no group activities were conducted as part of data collection for this study.

### Data Validation Workshop

A rapid analysis of data was conducted and presented to key South Sudanese stakeholders to assist with interpretation of the findings. Many of these stakeholders also provided reviews of the vignettes and question guides to ensure suitability for the South Sudan context.

## RESULTS

### Social Norms Related to Contraceptive Use

#### Pronatalism

Social norms in South Sudan are pronatalist, encouraging the practice of having as many children as possible and viewing children as God’s desire for them to reproduce. Participants reported that it was culturally appropriate to expect married women to fulfill their biological roles by bearing children, and this was linked to Christian directives in the Bible.

*That is what we were created to do, we are supposed to bring children to this world. You are expected to produce children.* —Married woman, aged younger than 25 years, urban Budi County

*A woman is supposed to produce many children for the family…that is why God gave her the ability to get pregnant and not the man.* —Male key influencer, rural Yambio County

*Contraceptive use is not supported….Even the Bible asks for the world to be filled with many people.* —Female key influencer, rural Leer County

Respondents also noted that having children was a community mandate for family and clan continuity. Some commented that having children was needed to replace men and women who were lost in war. Several respondents noted that because South Sudan has plenty of land it could accommodate a larger population, thus reinforcing the norm that large families were desirable.

*In South Sudan, people need to produce children in plenty; we have the land, and we need people to utilize it.* —Married man, aged older than 25 years, urban Bor County

*People keep dying, so the children produced replace the people dying.* —Female key influencer, rural Leer County

*We have lost a lot of people in South Sudan. We do not need contraceptives, and we need to repopulate our land.* —Male FP provider, rural Wau County

#### Patriarchy

Participants reported that men were expected to make major decisions as they were considered the head of the family. The woman needed his consent for most things, including FP.

*A man, being the head of the family, he must be responsible, take care of the family, and should make good decisions that benefit the family. . . . Even the Bible shows a man as the head of the family, and his decisions must be respected. —Married man,* aged older than 25 *years, rural Budi County*

*The man knows what is good for him and his family; if he says “yes” [to using contraceptives], then it is okay.* —Married man, aged older than 25 years, urban Bor County

Most respondents noted that reproductive choices and decision-making were controlled by men. They noted that FP/RH decisions had implications for the family, clan continuity, and the community. FP/RH health care providers also noted the importance of male partner consent for contraceptive use. Many reported an unwritten rule that in some health facilities, FP services could be provided only in the presence of a male spouse.

Most respondents noted that reproductive choices and decision-making were controlled by men.

*The woman is not supposed to do anything…without the man’s consent. If he finally agrees, then she can right away run to get the contraceptive method.* —Married man, aged older than 25 years, urban Wau County

*We cannot give contraception to her if she comes alone to the health facility, unless she comes with her husband.* —Female FP provider, rural Leer County

*I totally refuse [to provide a contraceptive] if the man has not accepted her to use [one].* —Female FP provider, urban Yambio County

Respondents stated that the father often determined when his daughter got married and whom she married. Early marriage is common in South Sudan. Marriage is viewed as transactional; the daughter is considered a commodity that would bring in money and resources. The bride price (dowry) paid to the bride’s family assumed payment to produce many children. Moreover, if married early, the bride would still be expected to become pregnant shortly after marriage, which translated into girls getting pregnant during adolescence.

*The father decides on when this [to marry off his daughters] shall happen, and no one can object to his decision.* —Male key influencer, urban Bor County

*The mother went through [a] similar experience. . . . She cannot stand against the idea of having her [daughter] married off.* —Married man aged older than 25 years, rural Bor County

*His family will be upset…because they have taken cows from her husband with the hope that she will produce kids for him.* —Female key influencer, Bor County

Respondents noted that many men believed that women who used contraceptives were engaging in extramarital affairs. Respondents claimed that men’s decision against contraceptive use was not always about childbearing but rather preventing the possibility of having sex outside of marriage.

*You know, contraceptive use makes men feel insecure. The husband feels that the woman will now start going out with other men. So, the men are against contraceptive use because they want to prevent their women from going out with other men. There is nothing else*. —Married woman, aged older than 25 years, rural Yambio County

*When you provide pills or implants or any other contraceptive to the woman, some men might think that we are having sex with their wives.* —Male FP provider, urban Yambio County

#### Influencers: Family and Community

Respondents reported that a couple’s family and community also greatly impacted decisions around FP. Often, they were the gatekeepers of social norms, enforcing norms that they felt should be adhered to and affecting modifications to these norms. The main influencers included parents, male and female traditional authorities, religious leaders, peers, and health care providers.

*Children love and listen to their parents more than any other person…Most parents do not allow anyone outside the family to advise their daughter, as they think…[they] may provide wrong advice on contraceptive use, which could have devastating side effects on their child.* —Male key influencer, urban Wau County

*People look at it [FP] as something a woman discussed with her man, but this also involves him asking for the opinion of the family. Remember, what they are talking about is about expanding the clan.* —Local chief, rural Yambio County

*Community leaders can advise on health issues in the community, and other elders are also able to add a voice.* —Male FP provider, rural Leer County

*Community members have an upper hand in making decisions on contraceptive use because the woman and all the children she produces belong to the community.* —Male key influencer, urban Bor County

#### Gender-Based Violence Associated With the Use of Contraceptives

Respondents noted that female agency to use contraceptives and engage in FP could make women and girls susceptible to physical and psychological GBV. Use of contraceptives without her husband’s consent could put the woman at risk of physical assault, threats, verbal abuse, and/or divorce. Some noted that they knew several married men who encouraged other men whose spouses used contraceptives to take another wife. These threats and abuse did not only occur within the couple but also came from family members and gatekeepers. Some remarked that local chiefs were known to punish women who used contraceptives without their spouse’s consent. Participants also noted stigma associated with women thought to use FP, as they could be labeled promiscuous and/or witches.

Women’s use of contraceptives without her husband’s consent could put the woman at risk of physical assault, threats, verbal abuse, and/or divorce.

*Producing children is God’s plan, and if the…woman had decided to take contraceptives…I am obliged to punish that woman…usually 6 months of labor at my home, and also the service provider provides 3 months of labor at my home.* —Local chief, rural Yambio County

*Going for FP services without informing the husband is compared to being a witch in the community. This disappoints the husbands, family members, neighbors, and the entire community.* —Female key influencer, rural Budi County

#### Adolescents and Family Planning/Reproductive Health

Respondents noted that the norm was that a girl should remain a virgin until she was married. Stakeholders who participated in the data validation workshop noted that if a girl was thought not to be a virgin, it could lower the bride price and bring judgment against her and her family. They also noted if she was known to use contraceptives, this would lower the bride price.

Study participants reported that if a girl was thought to be too young to begin menstruating, she could be stigmatized as promiscuous. In some communities, respondents stated that when a girl began menstruating, men in her family assumed she had lost her virginity and may beat her.

*When a woman gets menses for the first time, it shows that she has lost her virginity and has started moving out with men. —Local chief, rural Yambio County*

*The girls also do not tell their fathers that they are menstruating. The fathers may think that…she has lost her virginity and usually they beat her up.* —Married woman, aged older than 25 years, rural Yambio County

*If her brothers come to know about this [sister’s menses], the brothers usually beat her, in view that she has started sleeping with men.* —Married man, aged older than 25 years, rural Yambio County

*Girls experiencing menses for the first time before the age of 15 are mocked by their older friends and labeled as prostitutes.* —Unmarried woman, aged younger than 25 years, urban Leer County

#### Risks to Family Planning/Reproductive Health Providers

FP health providers noted that they felt they were at risk of threats and abuse if they provided FP services and contraceptives to a woman without her husband’s consent. These threats and abuse did not only come from the husband. Some remarked that local chiefs were known to punish FP providers who offered services without male consent.

*If the husband discovers that his wife is taking contraceptives, he can sue both the wife and the doctor in local court; [the doctor can] be arrested because they are stopping the wife from producing more children for the family. . . . They can also be fined with very many cows.* —Married man, aged older than 25 years, rural Leer County

*Men are the ones who scare us, the service providers, from giving their wives contraception, because if you do so, the husband might come to harm you. We have encountered such problems several times.* —Female FP provider, rural Yambio County

*Two years ago, a midwife gave contraceptives to a woman. One day, her husband found the card in her purse. The man fought with her [his wife] and he came to that midwife saying, “You are the one supporting my wife to do prostitution.” The midwife was charged 20,000 South Sudanese pounds.* —Male FP provider, rural Wau County

*We call them medical dilemmas; this is where the client needs the services, but providing it might cause more harm to her and the service provider. You end up deciding that one should not access this service, and in the end, our work ethics are compromised. —*Female FP provider, urban Bor County

*They often say, “Should I hear anyone or a health provider giving contraceptives to my wife or to my child, I will come and blow off the head.”* —Male FP provider, urban Wau County

### Emerging Supportive Social Norms

It is important to also consider social norms that support the use of FP. Some participants identified that the benefits of modern contraceptive use included birth spacing, reducing unintended pregnancies, controlling family size, improving child health, having the ability to invest in a child’s well-being, and continuing school.

#### Women’s Agency

Social norms in South Sudan are shifting, giving women more agency and voice within society and exercising more self-determination. Some respondents in urban payams reported being able to exercise their agency to negotiate access to FP services without the consent of their husbands. Some older married women (aged 25 years or older) and FP/RH providers explained that this was the right thing to do, given that the burden of care often lay with women. Some respondents explained that the effect of unplanned pregnancies on the body was experienced by a woman, and therefore, she held the ultimate decision-making responsibility.

*Women are the ones who understand their bodies and know what is happening to them. The husbands do not know what happens in a woman’s body.* —Male FP provider, urban Yambio County

*At times, it’s too much for us women. We decide that we need a break, whether he agrees or not. If he does not agree, then he leaves me with no choice but to use it without his consent.* —Married woman, aged older than 25 years, urban Budi County

*We are always told that we should give birth to children. This is easy to say, but for those of us who have gone through it, we know what it means. It reaches a point when you cannot take it anymore, and you have to use them [modern contraceptives] whether he likes it or not.* —Married woman, aged older than 25 years, urban Leer County

Some women reported seeking FP services secretly for unmet need if they believed that they would not be supported by their spouse, family, and/or community.

*They [women] come when the regular outpatients have left the hospital. . . . They do not want their identities disclosed to other people.* —Female FP provider, urban Bor County

#### Birth Spacing

Across most respondent groups, postpartum abstinence was a commonly held social norm, mainly to support the child’s health and well-being. Participants in Wau, Bor, and Budi counties reported that married couples should abstain from sex for 2–3 years after childbirth to achieve spacing between pregnancies. A community leader from Budi County reported that men who failed to abstain from sex with wives who recently gave birth were mocked, laughed at, and shamed. However, respondents also noted that women tended to uphold this norm, and men may abstain from sex with their wives but may take a second wife or find another sexual partner during this time.

Postpartum abstinence was a commonly held social norm, mainly to support the child’s health and well-being.

*She wants to space her children…continuous births bring about problems. Sometimes if the woman conceives when the other baby is still young, it might die because of improper care.* —Unmarried woman, aged older than 25 years, urban Wau County

*She wants to have child spacing that may help her to manage her family and put her 2 children into good schools. Family planning is the best way to achieve child spacing and manage a family.* —Unmarried man, aged older than 25 years, urban Leer County

*One has to abstain from sex for 2 to 3 years after [the] birth of a child. . . . Communally, a man is not allowed to have sex with his wife during this period, for those who tempt to have sex before 2 years elapse, they are mocked, undermined, and discouraged by the society through community talks and locally composed songs that humiliate and shame those men.* —Female key influencer, rural Budi County

#### Education

The importance of education, especially for girls, was mentioned by several respondents, particularly by those living in urban settings, as well as younger adults and some FP providers. They emphasized the role of contraceptives in school completion.

*I will tell her, my daughter, “Producing too many children…is also not good…contraception…can protect yourself…so that [my daughter]…can join school. —*Married woman, aged older than 25 years, urban Yambio County

*It is best if she first finishes school; then she will be able to look after her family. —*Married man, aged older than 25 years, urban Budi County

*They can come saying that they want to marry his daughter and he will tell them that they let her first study; they should come back when she has completed school. —Female key* influencer, urban Wau County

#### Delaying Early Marriage

Younger respondents and those from urban settings questioned some of the traditional social norms more often and were more supportive of the use of modern contraceptives. For example, although early marriage is common, some began to question this practice.

*Early marriage is strongly discouraged; it should be 18 and above, and the chiefs punish according to the laws. But if the case is beyond the capacity of the chief, he refers it to the big court in Wau.* —Male key influencer, urban Wau County

### Patient-Provider Communication on FP/RH

Using a set of 5 structured statements, community respondents, key influencers, and FP/RH providers were asked about patient-provider interactions related to FP/RH services.

#### Community Perspectives

Overall, most community respondents felt that “Rose,” the central protagonist in the vignettes, could talk to her provider, initiate conversations, ask questions, and share opinions about using contraception and that the provider pays attention to what Rose has to say (Table 4). Among those who disagreed with these 5 statements, the percentage of men disagreeing was approximately twice that of women.

**TABLE 4.** Community Respondent Perspectives on Patient-Provider Communication on Family Planning and Reproductive Health, South Sudan

	**No. (%)**		
	**Agree**	**Disagree**	**Do Not Know**
1. Rose and her health care provider talk about using contraception.			
All (N=233)^a^	148 (63.5)	60 (25.8)	25 (10.7)
Female (n=129)	86 (66.7)	21 (16.3)	22 (17.1)
Male (n=104)	62 (59.6)	39 (37.5)	3 (2.9)
Urban (n=122)	85 (69.7)	28 (23.0)	9 (7.4)
Rural (n=111)	63 (56.8)	32 (28.8)	16 (14.4)
2. Rose can initiate conversations about using contraception with her health care providers.			
All (N=233)	145 (62.2)	61 (26.2)	27 (11.6)
Female (n=129)	84 (65.1)	22 (17.1)	23 (17.8)
Male (n=104)	61 (58.7)	39 (37.5)	4 (3.8)
Urban (n=122)	81 (66.4)	30 (24.6)	11 (9.0)
Rural (n=111)	64 (57.7)	31 (27.9)	16 (14.4)
3. Rose can ask her health care provider questions about using contraception.			
All (N=233)	163 (70.0)	40 (17.2)	30 (12.9)
Female (n=129)	93 (72.1)	15 (11.6)	21 (16.3)
Male (n=104)	70 (67.3)	25 (24.0)	9 (8.7)
Urban (n=122)	93 (76.2)	20 (16.4)	9 (7.4)
Rural (n=111)	70 (63.1)	20 (18.0)	21 (18.9)
4. Rose can share her opinions about using contraception with her health care providers.			
All (N=233)	163 (70.0)	38 (16.3)	32 (13.7)
Female (n=129)	90 (69.8)	14 (10.9)	25 (19.4)
Male (n=104)	73 (70.2)	24 (23.1)	7 (6.7)
Urban (n=122)	92 (75.4)	19 (15.6)	11 (9.0)
Rural (n=111)	71 (64.0)	19 (17.1)	21 (18.9)
5. When discussing contraception with her health care provider, she/he pays attention to what Rose has to say.			
All (N=233)	180 (77.3)	22 (9.4)	31 (13.3)
Female (n=129)	97 (75.2)	7 (5.4)	25 (19.4)
Male (n=104)	83 (79.8)	15 (14.4)	6 (5.8)
Urban (n=122)	99 (81.1)	12 (9.8)	11 (9.0)
Rural (n=111)	81 (73.0)	10 (9.0)	20 (18.0)

^a^ One female respondent did not answer the structured Likert questions. Total community and influencer respondents on the structured questions was 233.

#### Family Planning Provider’s Perspectives

FP/RH providers were asked similar questions about patient-provider communication and stated if they agreed or disagreed (none answered that they did not know). Most agreed with the statements supporting communications related to FP/RH (Table 5). Although there were some differences between male and female respondents and rural and urban respondents, the total number of provider respondents (N=32) was too small to apply meaning to these differences among those who disagreed with the statements.

**TABLE 5.** Health Care Provider Perspectives on Patient-Provider Communication on Family Planning and Reproductive Health, South Sudan

	**No.(%)**	
**Respondent**	**Agree**	**Disagree**
1. Unmarried young women (age 14–16 years) and their health care provider talk about using contraception.		
All (N=32)	25 (78.13)	7 (21.88)
Female (n=15)	11 (73.33)	4 (26.67)
Male (n=17)	14 (82.35)	3 (17.65)
Urban (n=14)	12 (85.71)	2 (14.29)
Rural (n=18)	13 (72.22)	5 (27.78)
2. Married young women (age 14–16 years) and their health care provider talk about using contraception.		
All (N=32)	26 (81.25)	6 (18.75)
Female (n=15)	12 (80.00)	3 (20.00)
Male (n=17)	14 (82.35)	3 (17.65)
Urban (n=14)	12 (85.71)	2 (14.29)
Rural (n=18)	14 (77.78)	4 (22.22)
3. Adult women and their health care provider talk about using contraception.		
All (N=32)	29 (90.63)	3 (9.38)
Female (n=15)	15 (100.00)	0 (0.00)
Male (n=17)	14 (82.35)	3 (17.65)
Urban (n=14)	12 (85.71)	2 (14.29)
Rural (n=18)	17 (94.44)	1 (5.56)
4. Women can initiate conversations about using contraception with their health care.		
All (N=32)	29 (90.63)	3 (9.38)
Female (n=15)	15 (100.00)	0 (0.00)
Male (n=17)	14 (82.35)	3 (17.65)
Urban (n=14)	12 (85.71)	2 (4.29)
Rural (n=18)	17 (94.44)	1 (5.56)
5. Women can share their opinions about using contraception with their health care.		
All (N=32)	32 (100.00)	0 (0.00)
Female (n=15)	15 (100.00)	0 (0.00)
Male (n=17)	17 (100.00)	0 (0.00)
Urban (n=14)	14 (100.00)	0 (0.00)
Rural (n=18)	18 (100.00)	0 (0.00)
6. When discussing contraception with her health care provider, he/she pays attention.		
All (N=32)	31 (96.88)	0 (0.00)
Female (n=15)	15 (100.00)	0 (0.00)
Male (n=17)	16 (94.12)	0 (0.00)
Urban (n=14)	13 (92.86)	0 (0.00)
Rural (n=18)	18 (100.00)	0 (0.00)

## DISCUSSION

This study identified restrictive and supportive social norms related to FP/RH. Findings suggest that norms shape the dominant social practices^22^^,^^28^^,^^29^ surrounding modern contraceptive use. Key influencers reflected the dominant community attitudes, beliefs, and practices and, therefore, reinforced existing community, social, and gender norms.^30^ Social norms articulated by study respondents are imbued with various community values^31^ related to male privilege, family honor, fertility, and collectivism,^32^ among others.

Social norms present expected behavior patterns^33^ on the use of FP/RH services and contraceptives. Some patterns observed in this study reflect entrenched, dominant gender roles, such as male consent needed to use contraceptives.^4^^,^^34^ This also reflects the power differentials within society between men and women,^35^^,^^36^ which reflect hegemonic masculinity. This undermines the goals of the International Conference on Population and Development (ICPD), focusing on the needs, aspirations, and rights of women and men.^37^^,^^38^ This hegemonic masculinity underpinned by existing patriarchal norms limits women’s and girls’ agency to translate behavioral intentions for FP use into practice. Women’s limited agency related to FP is also linked to male authority connected to making decisions about when a girl marries, and female commodification as demonstrated by family income linked to her bride price.^39^^,^^40^ Furthermore, the implication is that there are risks for a woman and her reputation if she does not comply with these existing social norms.^22^

Gender and social norms can contravene the RH rights of women and adolescent girls. If a woman does not adhere to the expected social norms related to FP/RH, she may be ridiculed, threatened, and become a victim of violence. Her FP/RH provider is also at risk. As Mkandawire et al. describe,^9^ FP can be considered a foreign concept meant to destroy the local culture and population. Therefore, the spouse, family, and community are likely to push back to support existing social and gender norms.^39^^,^^41^ This South Sudan study revealed social norms and related sanctions that render it acceptable to engage in GBV to enforce adherence to the nonuse of modern contraceptives. The expectation that various sanctions are to be enacted following transgressions of particular norms, such as those related to discouraging FP use, normalizes women’s experience of various forms of violence and coercion.^32^^,^^40^ Moreover, this presents perpetrators with a “right” to inflict various forms of GBV on women and adolescent girls. It also builds social relationships characterized by women’s and girls’ victimization^32^ and involves the victimization of those providing FP services to women and girls without the consent of men and parents.

Some respondents noted the benefits of contraceptive use, particularly for the health and well-being of their children and to keep girls in school. Supportive social norms included abstinence from sex for at least 2 years after birth, the importance of education and school completion for girls, and emerging women’s agency, which reflects a contestation of some of the harmful social norms.^42^ Sexual abstinence for 2 to 3 years after birth, particularly in Wau, Yambio, and Budi counties, was seen as applying primarily to women, as it was communicated that men may engage in sex with other partners during this time period. About 41% of South Sudanese marriages are polygamous,^3^^,^^43^ with men having multiple wives. However, the norm that supports child spacing can be promoted to support the use of contraception to allow the practice of child spacing without requiring abstinence.

Although the South Sudan law states that the marrying age is 18 years and older, it is rarely enforced. Many respondents, especially those from urban settings, expressed the importance of girls staying in school and that contraceptive use may help delay pregnancies and foster school completion. Although school completion was seen as important, they reported disparate norms, such as beliefs (reported by many respondents) that the onset of menstruation signaled that a girl was ready for marriage; that her marriage would increase the family’s resources by way of payment of the bride price; and that, in some locations, menses onset meant a girl’s loss of virginity. School completion for girls reflects exercising agency that promotes gender equality with implications for reproductive health rights.^42^

Of significance is the norm of emerging women’s voice and agency, which shows progress toward the goals of ICPD.^38^ Some women could negotiate their reproductive choice with their partners and participate in planning their families. However, the study found that women’s input tends to reinforce communal norms, leading them to agree with reproductive decisions made by their husbands, families, and community.^22^ Another form of agency and resistance to social norms could be observed when a woman or girl sought FP/RH services secretly, often going to a clinic after hours or when she knew no one would see her there. However, such forms of agency have not achieved community-wide change in modern contraceptive use, with women’s agency constrained by structural factors, including limited availability and access to FP services. Such factors further limit one’s capacity to mobilize against restrictive norms. Other scholars have noted that social norms transformation has to be combined with other interventions that address structural barriers to access to FP/RH services to make it effective in promoting the use of these services.^29^^,^^44^

Some women could negotiate their reproductive choice with their partners and participate in planning their families.

To reduce inequalities in access to FP/RH universally, it is essential that programs working to promote FP in South Sudan consider the challenges presented by social norms and the risk to the women and their FP/RH providers. FP programming often addresses gender inequities but does not consider the risk presented by existing, incompatible social norms. An FP/RH program in South Sudan should build upon and expand supportive and emerging social norms while making sure they do no harm.

The use of vignettes led to a more comprehensive understanding of the social norms associated with FP/RH, as well as the risk of GBV, that a survey or semistructured interviews would not have achieved. Although these findings are related to select counties in South Sudan, they may have implications for FP/RH programming in other sites as well. Minimally, the use of vignettes to explore social and gender norms among other populations and other sensitive topics is recommended.

Findings from this study were shared with stakeholders and the communities included in the study sample. The communities are now working with MOMENTUM Integrated Health Resilience to develop and implement FP/RH programs that address constraints presented by existing social norms and that build on and strengthen supportive social norms. The approach has been to shift to a value proposition based on stronger families, healthier children associated with increased productivity, healthier women, and the value of male engagement. This value proposition embraces the focus on joint decision-making in the household on FP/RH, among other key health issues. The project’s SBC approach, developed with input from the communities and stakeholders, uses this value proposition approach. This process enhances localization and human-centered interventions. Communities, with support from MOMENTUM Integrated Health Resilience, are now implementing SBC and client-defined quality activities.

This dissemination of findings and community dialogues can lead to an organic movement of collective agency. Individuals and groups will then carry these findings and solutions toward social norm transformation to their families, social groups, and networks. As they gather in the market, at water pumps, churches, and other centers for meeting, they will informally generate new discussions and generate solutions through this snowball manner. Through this informal process of collective agency social norms transformation can occur, engaging women, youth, men, and all segments of the community. This is in line with the Program of Action that resulted from ICPD.^37^

## CONCLUSION

The use of vignettes to gain an understanding of the role social norms play in FP/RH proved to be very successful. This methodology allowed for study respondents to comfortably provide information on topics that are often sensitive and not discussed openly. It provided a wealth of information regarding social norms that MOMENTUM Integrated Health Resilience was able to use in concert with communities and stakeholders to strengthen FP/RH in South Sudan. These findings led to activities by the project and the communities that are in line with the Program of Action resulting from the ICPD meeting in Cairo 30 years ago.

In South Sudan, existing social norms are generally not supportive of FP/RH and particularly of women’s agency to control their own RH. If they seek FP/RH services or use contraceptives, women and their health care providers are at risk of coercion, threats, and violence, especially if their husbands have not given permission. However, there are emerging social norms that support FP/RH. For example, many respondents stated the importance of girls staying in school. This will delay early marriage and likely sexual debut. Given the need for male support for the use of FP/RH services and contraceptives, it is essential to support male engagement while also supporting women’s agency.

Social norms transformation can only occur through a collective or participatory approach. Individuals and collective groups must own the need for social norms transformation and the importance for women seeking FP/RH services and decide solutions aimed at increasing use of FP/RH.

A study of social norms identifies entrenched obstacles to FP/RH, as well as facilitating norms supportive of FP/RH. It is essential to the success of FP/RH projects, such as MOMENTUM Integrated Health Resilience, to understand the local context and social norms, and possible unforeseen consequences of their programming and interventions, particularly so they do no harm. Projects should also build on supportive social norms as they create a foundation for building, strengthening, and expanding the demand for and use of FP/RH.

## Notes

**Peer Reviewed**

**First Published Online:** June 24, 2024
